# Individual change in rejection of equal opportunities for foreigners among adolescents and young adults in Switzerland: Testing realistic conflict theory from a dynamic perspective

**DOI:** 10.1371/journal.pone.0296883

**Published:** 2024-02-07

**Authors:** Inge Hendriks, Marcel Lubbers, Peer Scheepers

**Affiliations:** 1 Department of Sociology, Radboud University, Nijmegen, The Netherlands; 2 ERCOMER / Interdisciplinary Social Science, Utrecht University, Utrecht, The Netherlands; Bar-Ilan University, ISRAEL

## Abstract

This study’s objective is to test one of the key theoretical orientations in the literature on intergroup relations, realistic conflict theory, from a dynamic perspective. In this study, we focus on adolescents who are, according to the ‘impressionable years’-hypothesis, most likely to change in their rejection of equal opportunities. Realistic conflict theory is applied in a dynamic way by taking into account economic characteristics related to the adolescent and the household. We employ Swiss panel data covering the period 1999–2017, containing information of both adolescents and the household they live in. Results demonstrate that there is relatively little variation within adolescents in rejection of equal opportunities for foreigners. Despite of using a broad range of characteristics related to adolescents’ as well as the household’s economic situation, the changes that do occur within individuals could hardly be explained by those economic changes. Socialisation theories on direct parental influences appeared to better explain changes in rejection of equal opportunities.

## 1. Introduction

One of the key theoretical approaches to intergroup relations is realistic conflict theory [1]. In short, realistic conflict theory argues that intergroup competition over scarce resources induces incompatible group interests and might consequently drive antagonism between groups in society [2, 3]. In the literature, much attention has been devoted to why and under which circumstances intergroup antagonism comes about [e.g. 4, 5, 6]. Up to now, little attention has been paid to the dynamic interpretation of realistic conflict theory, i.e. whether and why rejection of equal opportunities for foreigners changes within individuals. Obtaining a better understanding of the dynamics of intergroup attitudes is yet of particular interest when considering the way in which immigration rapidly changes the composition and face of European societies [7], coinciding with major economic fluctuations.

Only few studies have used panel data to study individual dynamics of intergroup attitudes. These studies, however, do not provide conclusive evidence for the dynamic version of realistic conflict theory. For instance, Lancee and Pardos-Prado [8] demonstrate that becoming unemployed makes people less likely to offer immigrants equal opportunities, but others [9–11] do not replicate this dynamic effect. Also, a change in individuals’ income was found to increase anti-immigrant sentiments to some extent [12], whereas other studies have found this effect to be not significant [9–11]. Moreover, evidence for the impact of changes in individuals’ perceptions, such as satisfaction with the economy and financial worries, is mixed as well [12–14].

Instead of adding another study testing these dynamic effects within the general population, this study takes on a different approach to get better insight in the dynamic interpretation of realistic conflict theory as we focus on adolescents in particular. Previous research demonstrated that intergroup attitudes are in general rather stable [12, 15, 16]. This lack of versatility in individuals’ intergroup attitudes could signify a need for specifying the premises of the dynamic version of realistic conflict theory. Typically, the dynamic version of realistic conflict theory presumes ‘lifelong openness’ to attitudinal change. This contrasts the notion of the ‘impressionable years’ mentioned in the psychological development literature, which states that adolescence and young adulthood is pivotal in the formation of attitudes [17–19]. By focussing on the individual change in rejection of equal opportunities for foreigners among adolescents and young adults, we contribute to the literature as we combine the social psychological and sociological literature on realistic conflict theory with assumptions retrieved from psychological studies on adolescents’ attitude formation.

Recently, Lancee and Sarrasin [20] have focused on changes in intergroup attitudes related to realistic threat among adolescents. Following other studies on the pivotal role of education in understanding negative attitudes towards immigration [21, 22], they examine the liberalising effect of education and find that adolescents become more negative towards immigrants as they enter the labour market. Also, their analyses demonstrate that changes in satisfaction with the financial situation appeared to influence adolescents’ attitudes towards immigrants.

We aim to innovate upon recent studies in order to acquire more insights in dynamic effects among adolescents. First, we propose a broader theoretical framework of the dynamic version of realistic conflict theory considering crucial economic transitions that adolescents go through in this particular phase in their lives. Second, we do not only take adolescents’ individual economic transitions into account, but also changes in objective and subjective household characteristics in which adolescents grow up. Taking into account the household context when testing realistic conflict theory among adolescents is particularly relevant as the economic context in which adolescents come of age is mainly determined by the household. Third, when testing whether economic transitions in the household affect adolescents’ rejection of equal opportunities, we also include parental socialisation, i.e. parents’ rejection of equal opportunities. By including and theorizing about this broad range of adolescent-specific and household-specific (economic) factors, we improve upon previous studies [e.g. 8, 20, 23] that focused on single changes in explaining intergroup attitudes.

In short, the objective of this study is to test the dynamic version of realistic conflict theory for those who are most likely to change in rejection of equal opportunities for foreigners according to the ‘impressionable years’-hypothesis, i.e. adolescents. We employ Swiss panel data of fourteen waves covering the period 1999–2017, containing information of both adolescents and their parents. These data do not only allow for testing whether economic transitions in individuals’ life as well as changes in the household context evoke a change in rejection of equal opportunities, but also enable us to model to what extent adolescents’ and parents’ rejection of equal opportunities for foreigners change concurrently.

## 2. Theoretical framework

### 2.1 Adolescents’ changing rejection of equal opportunities

According to realistic conflict theory, intergroup antagonism can be explained as a result of competition between social groups in society over scarce resources [2, 24–26]. Because of intergroup competition over scarce resources, incompatible group interests arise which are argued to enhance solidarity within a social group and induce antagonism between social groups in society (see [1] for more information). The likelihood to experience intergroup competition is assumed to be particularly prevalent among those whose socio-economic position resembles the position of out-group members, i.e. individuals who find themselves in the less favourable socio-economic strata of society [27, 28].

The dynamic version of realistic conflict theory does not assume that individuals have “permanent hostile attitude[s] toward the outgroup based on innate dispositions, authoritarian personalities, or slow social experiences” [8, p. 109], but proposes more flexibility and versatility of intergroup attitudes. This means focus is placed on differences in intergroup attitudes *within* individuals instead of differences *between* individuals [7, 11]. Hence, the general proposition of the dynamic perspective on intergroup competition argues that rejection of equal opportunities for foreigners increases when individuals’ socio-economic status deteriorates to a level that they are more strongly in competition with out-group members.

We add insights from the psychological development literature to this dynamic version of realistic conflict theory, stating that attitudes are especially prone to change during adolescence and young adulthood. Though there is no clear consensus about precisely which years people are most likely to change in their attitudes, this study follows other studies that argue that political socialisation still takes place up to the mid-twenties [15, 19]. According to the ‘impressionable years’-hypothesis, adolescence is pivotal in the formation of attitudes [19, 29]. Later in life, these attitudes become ‘crystallised’, indicating that individuals are then less vulnerable to attitudinal change. Such ‘crystallisation’ of intergroup attitudes is confirmed by for instance Henry and Sears [17], who demonstrate that most within-individual change in symbolic racism is observed during people’s early adulthood. Since the literature refers to different ages to describe the impressionable years, we include the population between 14 and 25 in this study, to which we refer as the adolescent and young adult population. We therefore expect that the dynamic version of realistic conflict theory is in particular applicable to adolescents and young adults. Therefore, our specific proposition is that primarily among this group rejection of equal opportunities is enhanced by a transition towards a position in which competition with ethnic out-groups is more likely.

A key transition during adolescence that is likely to increase experienced competition with ethnic outgroups is entering the labour market [20]. One of the main scarce resources that creates incompatible interests between social groups in society is jobs [1, 24]. Competition over jobs and economic privileges may create fear or anxiety towards ethnic outgroups, as they can undermine the natural superiority of the dominant group by claiming these scarce resources [30]. Hence, as adolescents complete their education and enter the labour market, they are expected to find themselves in a situation of competition with ethnic outgroups over jobs. Due to this higher level of competition, adolescents might become less supportive of equal opportunities for foreigners. Considering that especially those who are not able to find a job are vulnerable to experience competition from foreigners [31], we expect that entering the labour market but not being able to find a job increases the likelihood to reject equal opportunities even more. This results in the following hypothesis:

H1: *(a) As adolescents enter the labour market*, *the likelihood to reject equal opportunities for foreigners increases*, *(b) and this is in particular the case when adolescents are not able to find a job*.

Before completing education, some adolescents may already become acquainted or have a first encounter with the labour market. This holds primarily for adolescents who follow vocational education with high labour market relevance. Adolescents who receive vocational or professional training with special focus on labour market competences and who do work placement are more likely to get a sense of intergroup competition with ethnic outgroups already at an early stage of their career, which is expected to evoke an increase in anti-immigrant attitudes such as rejection of equal opportunities [20]. Previous research, furthermore, demonstrated that adolescents in vocational education are more likely to experience an increase in ethnocentrism over time compared to their counterparts in general education [32]. We therefore expect that:

H2: *As adolescents start with vocational training*, *the likelihood to reject equal opportunities for foreigners increases*.

Not only objective, tangible transitions in adolescents’ life can evoke a change in rejection of equal opportunities. Previous research [e.g. 12, 33] has demonstrated that perceptions of a worsened socio-economic position might also spark rejection of equal opportunities, independent of the influence of objective transitions in life. Blalock’s [2] distinction between actual and perceived competition helps us to understand the influence that independent, subjective changes can have on the change in rejection of equal opportunities. Though actual competition may be absent, individuals who perceive a deterioration in their socio-economic position can still have a higher level of perceived competition, which might in turn spark rejection of equal opportunities for foreigners [12, 34]. Hence, we expect that:

H3: *As adolescents’ subjective economic dissatisfaction increases*, *the likelihood to reject equal opportunities for foreigners increases*.

### 2.2 The impact of the economic household context

Childrens’ attitudes can be influenced by their parents in multiple ways. This might hold in particular for adolescents and young adults who still live with their parents and who are not economically independent yet. In this study, we focus on direct intergenerational transmission of attitudes within a family (see below) versus indirect transmission via parents’ (economic) position. The latter is called ‘positional parental socialisation’ [35], and underlines the impact of parents’ and childrens’ shared environement and common social status. Adolescents are exposed to divergent household experiences, because of differences in the social position of their parents and the household they live in. Hence, it is argued that indirect parental transmission occurs via the provision of the setting in which children develop attitudes [36].

Considering that the household context is the main economic context in which adolescents come of age, we argue that adolescents are not only influenced by transitions and changes in their own economic situation but also by changes in the economic situation of the household that they live in. Previous research demonstrates that the structural and cultural position of the household matters to adolescents’ rejection of equal opportunities [37]. Growing up in relatively favourable economic living conditions is associated with lower levels of perceived ethnic threat and, subsequently, more favourable intergroup attitudes [38, 39]. Disadvantageous living conditions, on the other hand, can cause perceptions of disstress among members of the household [40], which may affect parents’ and adolescents’ level of perceived or experienced competition and, subsequently, their intergroup attitudes. When combining the notion of ‘positional parental socialisation’ with the theoretical assumptions of realistic conflict theory, we expect that economic characteristics of the household have an impact on adolescents’ attitude towards equal opportunities for foreigners.

From a dynamic perspective, we argue that a deterioration of the economic situation of the household implies less favourable living conditions, which can subsequently evoke higher levels of experienced or perceived competition. These higher levels of competition experienced from foreigners can in turn increase rejection of equal opportunities among all members of the household. As adolescents are especially prone to attitude change, we expect these negative changes in the household’s living conditions to increase rejection of equal opportunities among adolescents in particular.

One of the key characteristics that ensures an economically more secure position is a household’s income. Compared to their less privileged counterparts, households with a higher income are to a higher extent insulated from economic pressures induced by foreign competitors, for instance on the labour market [41, 42]. When experiencing a decrease in income, the economically secure and priviliged position of the household becomes pressured. Consequently, these pressures might evoke experiences of ethnic threat and increase rejection of equal opportunities. Based on studies accentuating the effect of the shared economic position [35, 36], we expect that a decrease in income has an effect on all members of a household, adolescents included. Therefore, we expect that:

H4: *If the household income decreases*, *adolescents’ likelihood to reject equal opportunities for foreigners increases*.

Another economic change that could have a large impact on the economically secure position of the household concerns a transition to unemployment of (one of) the household’s main earner(s). Being confronted with unemployment is often unforeseen and involuntarily, and can disrupt a households’ accustomed way of living [8, 40]. A transition to unemployment could lead to an increase in experienced ethnic competition, as one of the household members suddenly has to compete with outgroups on the labour market over jobs at offer [43]. The confrontation with unemployment and increased level of competition among the unemployed household member(s), can place the household and all of its members in an economically stressful position. These expectations lead to the following hypothesis:

H5: *If the household is confronted with unemployment*, *adolescents’ likelihood to reject equal opportunities for foreigners increases*.

Again, we expect that not only objective and tangible transitions in the household can lead to a change in rejection of equal opportunities, we also argue that perceptions of a worsened socio-economic position in itself can evoke rejection of equal opportunities [33]. If members of a household, parents in particular, perceive an increase in subjective economic dissatisfaction, a feeling of economic insecurity can be generated and discussed within the household. Though actual competition may be absent, household members could still perceive higher levels of competition as a consequence [2]. Because such subjective economic dissatisfaction creates a less favourable living environment in a household, we expect that:

H6: *If the household’s subjective economic dissatisfaction increases*, *adolescents’ likelihood to reject equal opportunities for foreigners increases*.

Not all households may be equally affected by changes in the economic situation. In general, foreigners in Switzerland have a less advantaged socio-economic status as compared to the native population [44]. Therefore, we expect that especially those who live in a household that is already positioned in the relatively lower socio-economic strata of society are more likely to find themselves in a situation of competition over scarce resources with foreigners as a consequence of a deterioration in their household’s socio-economic status [2, 45]. We hence expect that if the household’s economic position resembles the position of foreigners to a greater extent, an experienced or perceived change in the economic consequences has a greater impact on the likelihood to become less supportive of equal opportunities for foreigners. This results in the following hypothesis:

H7: *The effects of changes in a household’s economic situations on the change in adolescents’ likelihood to reject equal opportunities for foreigners (H4-H6) are stronger for those living in a household with a more disadvantageous socio-economic position*.

In research studying the impact of the household context on adolescents’ intergroup attitudes, parents’ role as direct socialising agents is emphasised. Already in 1954, Allport [46] argued that children’s intergroup attitudes are shaped by their parents’ intergroup attitudes. Parents are argued to create a learning environment for their children, in which their children are taught basic social norms and values [47]. Children are subsequently suggested to imitate their parents via modelling by adapting to their parents’ attitudes, resulting in intergenerational transmission of intergroup attitudes [23, 48]. Though evidence for the existence of parental socialisation of intergroup attitudes is mixed, the meta-analysis of Degner and Dalege [49] shows that there is a medium-sized average association between intergroup attitudes of parents and their offspring, and that this effect is particularly visible among adolescents.

As we set out to test adolescents’ rejection of equal opportunities dynamically, we expect that a change in parents’ rejection of equal opportunities is associated with a change in adolescents’ rejection of equal opportunities. Whereas we presume that attitudes are less vulnerable to change after adolescence [19], we argue that the changes in parents’ rejection of equal opportunities that do occur may evoke a change in adolescents’ rejection of equal opportunities. Longitudinal studies on direct parental socialisation indeed found support for linked changes in parents’ intergroup attitudes to changes in their children’s intergroup attitudes [23, 39, 50]. We therefore hypothesise that:

H8: *If parents’ likelihood to reject equal opportunities for foreigners increases*, *adolescents’ likelihood to reject equal opportunities for foreigners increases as well*.

## 3. Data and operationalisations

### 3.1 Data

In this study, we tested our hypotheses for the Swiss case. Switzerland is a country with a relatively high share of immigrants, as about 26% of its population is foreign-born. A large part of this foreign-born population has a European background (OECD, 2021). After World War II, mostly unqualified manual workers migrated to Switzerland, followed by their families. Since the 1990s, immigration policies have become more restrictive for these labour migrants, while favouring higher educated European immigrants. Comparable to other west-European countries, immigration and integration have become highly politicized topics in the political and public debate, reflected in the success of the populist radical-right Swiss People’s Party [51, 52]. By contrast, Switzerland is not an EU-member and cannot be categorised easily nor clearly into one of the typical welfare regimes in the EU because of its rather fragmented welfare state [53].

The Swiss Household Panel, collected by FORS center, is used to test our hypotheses. More information on the data collection procedure and consent concerning participant recruitment can be found in the Swiss Household Panel User Guide [54] or at www.forscenter.ch. Because we use data that is publicly available, we needed no further approval of an institutional review board for the data collection. This longitudinal panel is based on a stratified, random sample of private households in Switzerland. Data were collected by means of three different questionnaires: a household grid questionnaire to assess the household composition; a household questionnaire, filled in by the reference person of the household; and an individual questionnaire, administered to all members of the household aged 14 years and older. Interviews were initially conducted telephonically only, but since 2010 face-to-face interviews and web-based questionnaires were available for those reluctant to participate (1.03% of the observations). Response rates were relatively high; about sixty percent of the approached people responded to the initial questionnaire and about eighty percent of the respondents participated in follow-up surveys. Those who attrite were somewhat more often younger people, males, and lower-educated people [54–56]. Refreshment samples were added to restore the representativity of the panel. For more information on the fieldwork approach, response rates and attrition, and data documentation, see Voorpostel et al. [54].

The data used in this study covers the 1999–2017 period. Data gathered in 2010, 2012, 2013, 2015, and 2016 were not taken into account in this study, as our dependent variable was not included in these waves. Because of our focus on adolescents and young adults, we only included observations of respondents aged 14 to 25. Respondents that we could select as having a non-Swiss nationality were not incorporated in this study. Analyses were performed on two samples: to test the first three hypotheses we included all adolescents; to test the impact of the household context we only included adolescents who lived with (one of) their parents. For the latter group, we matched for each separate survey year adolescents’ individual data to data of the household questionnaire and (if available) individual data of both the mother and father. After deleting observations with missing values on the dependent or independent variables and after deleting respondents who participated only once, our final sample consisted of 9,530 observations from 2,353 respondents. On average, respondents participated 4.05 times. Of this final sample, 2,125 respondents lived with (one of) their parents, of whom we have 8,948 observations.

### 3.2 Dependent variable

We used the question ‘Are you in favour of Switzerland offering foreigners the same opportunities as those offered to Swiss citizens, or in favour of Switzerland offering Swiss citizens better opportunities?’ to measure whether adolescents *reject equal opportunities for foreigners*. According to Pecoraro and Ruedin [51], this measurement of rejection of equal opportunities for foreigners can be understood as an unfavourable attitude towards foreigners more generally. Those who answered ‘in favour of better opportunities for Swiss citizens’ clearly surpass and reject the option to provide equal opportunities to foreigners and hence were ascribed a score of 1, whereas those who answered ‘in favour of equality of opportunities’ were ascribed a score of 0. A score of 0 was also ascribed to those who answered ‘neither’ (6.03% of the observations). Robustness checks in which the ‘neither’ category was dropped or when these respondents were ascribed a score of 1 instead of 0 did not yield substantially different conclusions (see”S1 Table”). The descriptive statistics (see “Table 1”) demonstrate that 28.10% of the observations in our sample were in favour of offering Swiss citizens better opportunities than foreigners.

**Table 1:** Descriptive statistics.

		Min	Max	Mean/proportion (all adolescents)	Mean/proportion (adolescents who live with their parents)
Support for equal opportunities					
	*Equal opportunities*	0	1	0.719	0.722
	*Better opportunities for Swiss*	0	1	0.281	0.278
Main activity					
	*In school*	0	1	0.733	0.761
	*Employed*	0	1	0.160	0.130
	*Unemployed*	0	1	0.008	0.009
	*Other*	0	1	0.016	0.014
	*Missing*	0	1	0.082	0.087
Education					
	*Inapplicable*	0	1	0.259	0.231
	*Primary*	0	1	0.137	0.144
	*Secondary*, *no Maturity*	0	1	0.017	0.017
	*Secondary*, *with Maturity*	0	1	0.154	0.163
	*Secondary vocational*	0	1	0.237	0.250
	*Tertiary vocational*	0	1	0.065	0.064
	*University*	0	1	0.114	0.113
	*Missing*	0	1	0.017	0.018
Financial dissatisfaction^1^		0	10	3.166	3.157
Income					
	*First decile*	0	1		0.069
	*Second decile*	0	1		0.090
	*Third decile*	0	1		0.090
	*Fourth decile*	0	1		0.094
	*Fifth decile*	0	1		0.094
	*Sixth decile*	0	1		0.094
	*Seventh decile*	0	1		0.091
	*Eighth decile*	0	1		0.094
	*Ninth decile*	0	1		0.095
	*Tenth decile*	0	1		0.097
	*Missing*	0	1		0.091
Parental unemployment					
	*No unemployed parents*	0	1		0.982
	*Unemployed parent(s)*	0	1		0.015
Financial dissatisfaction household^2^		0	10		2.544
Mother’s attitude					
	*Equal opportunities*	0	1		0.567
	*Rejection of equal opportunities*	0	1		0.284
	*Missing*	0	1		0.149
Father’s attitude					
	*Equal opportunities*	0	1		0.470
	*Rejection of equal opportunities*	0	1		0.199
	*Missing*	0	1		0.331
Household composition					
	*Adolescent living with two parents*	0	1	0.754	0.803
	*Adolescent living with one parent*	0	1	0.145	0.154
	*Adolescent living with partner and/or child*	0	1	0.014	
	*Adolescent living alone*	0	1	0.036	
	*Other household type*	0	1	0.051	0.042

Source: Swiss Household Panel (SHP), 1999–2017

N _all adolescents_ = 9,530 observations of 2,353 respondents

N _adolescents who live with their parents_ = 8,948 observations of 2,125 respondents

^1^ SD _between_ = 1.704, SD _within_ = 1.562 for all adolescents, SD _between_ = 1.697, SD _within_ = 1.577 for those who live with their parents

^2^ SD _between_ = 1.574, SD _within_ = 1.178 for those who live with their parents

### 3.3 Independent variables

To get an insight in whether rejection of equal opportunities changes as adolescents enter the labour market, we first created a variable that captures adolescents’ *main activity*. Each year, respondents were asked if they were currently studying at a school. Those who indicated that they were not, were considered as having left school and having entered the labour market. For these respondents, we checked what their actual occupation was. The eleven categories from which respondents could choose were reduced to three categories: employed, unemployed, and non-employed. The fourth category encompassed those who are in school. An additional fifth category was created for those with a missing value on this variable.

Those respondents who did not finish education yet were asked what their current type of education was. Respondents could choose between 17 categories that were recoded according to a coding scheme of Bergman et al. [57] to the six educational levels in Switzerland: primary education, secondary education without Matura (i.e. Swiss high school diploma), secondary education with Matura, secondary vocational education, tertiary vocational education, and university. Those who indicated to be in secondary or tertiary vocational education were, given the strong role of learning practical labour market competences and the importance of internships in these types of education, considered to be in *vocational training*.

We measured *adolescents’ subjective economic dissatisfaction* with the following question: ‘Overall how satisfied are you with your financial situation?’. Respondents could answer on an eleven-point scale ranging from 0 ("not at all satisfied”) to 10 ("completely satisfied"). We recoded the scale so a higher score denotes a higher level of dissatisfaction.

The *household income* was measured by the equivalised Swiss Conference for Social Welfare (SKOS)-scale, which takes into account the size and the composition of the household. For each separate year, we constructed deciles. For each of these deciles a dummy-variable was created, as well as an additional dummy-variable for respondents with a missing score.

Parents’ working status was used to indicate whether a household was confronted with *unemployment*. If either adolescents’ father or mother indicated to be unemployed, respondents got ascribed a score of 1. If both parents in the household indicated to be employed and/or that they were not part of the labour force, respondents got ascribed a score of 0.

The item used to measure the *household’s subjective economic dissatisfaction* is similar to the one measuring adolescent’s subjective economic dissatisfaction. Here, however, the reference person of the household was asked how satisfied (s)he is with the financial situation of the household. A higher score on the eleven-point scale indicated a higher level of dissatisfaction.

*Parents’ rejection of equal opportunities* was measured in the same way as adolescents’ rejection of equal opportunities. For both the mother and the father, a variable was created where those in favour of better opportunities for Swiss citizens were distinguished from those who did not. Additional dummy-variables were created for respondents who lived with (one of) their parents, but whose mother or father had a missing score on this variable.

### 3.4 Control variables

In our analyses, we controlled for the *household composition*. Here, we distinguished between adolescents living with two parents, adolescents living with one parent, adolescents living alone, adolescents living with a partner and/or child, and other household types (e.g. households with other related family members). We also control for the *year* of the survey.

### 3.5 Methods

To get an insight into the individual change in rejection of equal opportunities for foreigners, we plot predicted (population averaged) margins. We also present the percentage individuals who experienced a within-individual change in their rejection of equal opportunities and the proportion of individuals who remained stable between succeeding waves.

To test our dynamic hypotheses, we make use of logistic fixed effects models [58]. Fixed effects models enable us to investigate within-person variation in rejection of equal opportunities, as these models examine whether a change in individuals’ rejection of equal opportunities is related to changes in their economic situation. Because of its focus on within-individual variation, all unobserved time-invariant heterogeneity between individuals can be partialed out in fixed effects models, which contributes to the reliability of the estimated effects. Since fixed effects models rely on within-person variation only when estimating the effects of changes in the economic situation on changes in attitudes towards equal opportunities, the analyses are based on respondents whose attitude towards equal opportunities for foreigners changes over time. To minimise threats of identification, we make use of a large sample of as much as 14 waves. Though observations of transitions to unemployment were still rather infrequent, our strategy of pooling as much as 14 waves lowered the risk of power issues as we aimed at incorporating as much transitions as possible on both the dependent and independent variable(s) that we could analyse. Given the dichotomous character of our dependent variable, we use logistic fixed effects models that estimate the likelihood of having a negative attitude towards equal opportunities for foreigners as determined by its logistic cumulative distribution.

In the first logistic fixed effects model, we estimate the likelihood to reject equal opportunities for all adolescents aged 14 to 25. As we are primarily interested in the way in which attitudes towards equal opportunities are affected by *transitions* to the labour market or to vocational training during adolescence, we apply a modelling strategy in which we differentiate between origin and destination categories (for more information on this modelling strategy, see [59]). That is, we are primarily interested in for instance the transition from being in school to being employed, and not the other way around. We therefore transformed the variables measuring adolescents’ main activity and educational attainment into several variables capturing the transition of interest. In the second logistic fixed effects model, we estimate the impact of changes in the household context. As these changes can only be estimated for those who live with (one of) their parents, we excluded respondents who do not live with (one of) their parents from this model. A test for collinearity showed that there was no multicollinearity between the variables in our models.

Additionally, we performed our analyses separately for adolescents living in households that could be argued to be more likely to end up in a situation of ethnic competition as a result of a deterioration of the economic situation: adolescents who live in a household with a lower rather than higher income or whose parents have a lower rather than higher educational attainment. Adolescents were denoted as living in a household with a lower income when the mean income decile of the household was below the fifth decile, representing the respondents in the 40% lowest household incomes. Whether parents were considered having a lower educational attainment was based on the number of years needed to complete the highest level of educational achieved. Using education in years enabled us to calculate a more fine-grained mean score of both parents for each respondent. In case information of only one of the parents was available, we used the score of just that parent. Parents were denoted as having a lower educational attainment when they had completed their highest level of education achieved in 12 years or less, which can be translated to an ISCED (International Standard Classification of Education 2011) level of 3, i.e. an educational attainment of upper secondary education or lower [60]. Though this cut-off point might seem relatively high, it represents about 40% of the respondents as well. We performed multiple robustness checks where we examined whether our outcomes were affected by our choice for a specific cut-off point. In most cases, changing the cut-off point did not yield different substantial conclusions. If the cut-off points that we chose did affect our conclusions, we discuss the outcomes of these robustness checks in the results section.

## 4. Results

### 4.1 Descriptive results

**Fig 1:**
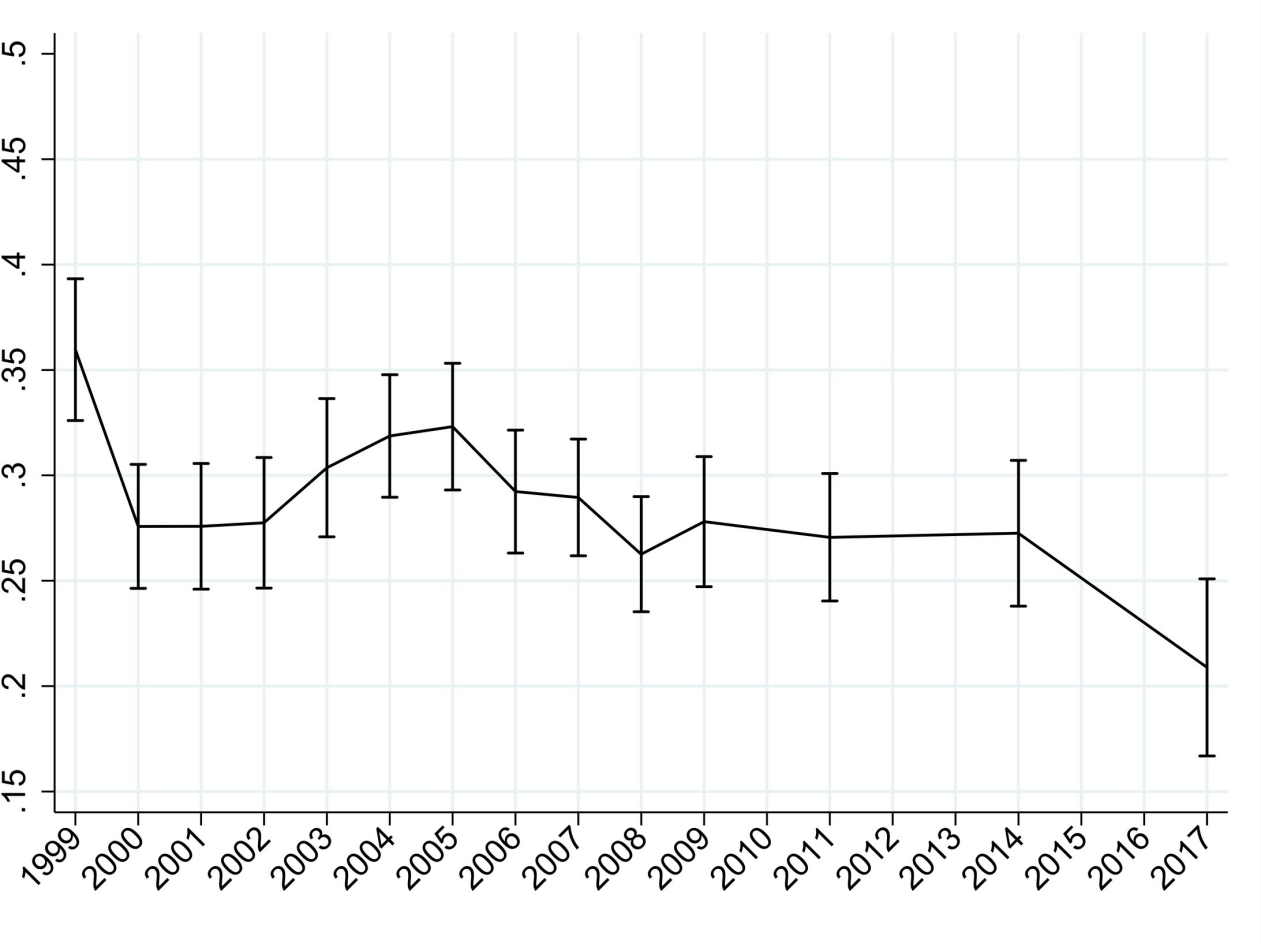
Margins plot on rejection of equal opportunities among adolescents. Source: Swiss Household Panel (SHP) 1999–2017, N = 9,530 observations of 2,353 respondents.

In “Fig 1” adolescents’ overall trend in rejection of equal opportunities among the members of the panel is presented. Rejection of equal opportunities was highest in 1999. Between 1999 and 2000, there is a decrease in rejection of equal opportunities for foreigners in general. From 2000 onwards, rejection of equal opportunities was found to be rather stable, as we could not observe significant changes in rejection of equal opportunities between succeeding years.

To get a better insight in the magnitude of change in rejection of equal opportunities among adolescents, we made an overview of the share of adolescents that experienced a within-individual change in rejection of equal opportunities for foreigners, as presented in “Table 2”. When comparing adolescents’ responses between succeeding waves, we see about 75% to 85% of the respondents remained stable in their rejection of equal opportunities. The individual change in rejection of equal opportunities was highest between 2000 and 2001; 23.39% changed in their rejection of equal opportunities during this period. Additional descriptive analyses furthermore showed that 66.52% of the respondents remained stable in their attitudes during all waves of participation. All in all, change in rejection of equal opportunities among adolescents thus is rather small.

**Table 2:** Percentage of adolescents that experiences a within-individual change in rejection of equal opportunities for foreigners between succeeding waves.

	1999–2000	2000–2001	2001–2002	2002–2003	2003–2004	2004–2005	2005–2006
Stable	77.30%	76.61%	81.39%	76.92%	83.70%	77.74%	81.00%
Change to rejection of equal opportunities	7.28%	11.79%	9.31%	13.68%	9.39%	11.82%	8.83%
Change to support for equal opportunities	15.42%	11.60%	9.31%	9.40%	6.91%	10.45%	10.17%
	2006–2007	2007–2008	2008–2009	2009–2011	2011–2014	2014–2017	
Stable	81.07%	82.94%	82.70%	81.06%	11.38%	80.74%	
Change to rejection of equal opportunities	8.93%	7.98%	9.37%	8.39%	9.54%	6.30%	
Change to support for equal opportunities	10.00%	9.08%	7.93%	10.55%	2011–2014	12.96%	

Source: Swiss Household Panel (SHP), 1999–2017

### 4.2 Multivariate analyses on changes in adolescents’ economic situation

The results of the logistic fixed effects models on the change in rejection of equal opportunities for foreigners are presented in “Table 3”. In the first model the effects of the individual characteristics are estimated for all adolescents. In this model we estimated the effect of the transition from school to employment or to unemployment on the likelihood to reject equal opportunities. Transitions from primary school to the labour market have been set to missing because of irregular patterns in the panel data. Our results indicate that the transition from school to employment induced a significant decrease in rejection of equal opportunities. This effect holds when controlling for household characteristics in the second model. For those who made the transition from school to unemployment we did not find a significant impact on the likelihood to reject equal opportunities. A closer look at this effect and its standard error demonstrates that the confidence interval of this effect is rather large (0.179 ± (1.96 x 0.447) = [-0.697; 1.055]). In comparison to the other estimates, the relatively high standard error and, accordingly, the rather wide confidence interval of the estimate for the transition to unemployment may indicate a relatively low power to test this effect. Indeed, only 46 respondents (0.48%) make the transition to unemployment. Based on these analyses, we therefore cannot support or refute our hypotheses on the transition to unemployment. For now, we can only state that finding a job decreases the likelihood to reject equal opportunities. Still, these findings are not in line with our first hypothesis that entering the labour market induced rejection of equal opportunities for foreigners among adolescents. In”S1 Text”, a more elaborate analysis can be found in which we estimated the effect of transitions from different educational levels to employment on changes in attitudes towards equal opportunities for foreigners. These additional analyses show that none of the specific transitions induce an increase in rejection of equal opportunities.

**Table 3:** Logistic fixed effects analysis on the likelihood to reject equal opportunities for foreigners.

		Model 1		Model 2	
		All adolescents		Adolescents who live with their parents	
		B	S.E.	B	S.E.
Labour market transitions					
	Transition to employment	-0.334 *	0.154	-0.319 *	0.160
	Transition to unemployment	0.179	0.447	0.229	0.455
Educational transitions					
	Transition to secondary vocational	0.080	0.136	0.064	0.139
	Transition to tertiary vocational	-0.677 **	0.234	-0.687 **	0.238
Financial dissatisfaction		0.020	0.020	0.023	0.021
Household income					
	*First decile*			ref.	
	*Second decile*			0.129	0.206
	*Third decile*			0.267	0.210
	*Fourth decile*			0.490 *	0.216
	*Fifth decile*			0.336	0.219
	*Sixth decile*			0.460 *	0.222
	*Seventh decile*			0.308	0.233
	*Eighth decile*			0.386	0.236
	*Ninth decile*			0.153	0.250
	*Tenth decile*			0.414	0.267
Unemployment parents				-0.457	0.354
Financial dissatisfaction household				0.039	0.028
Mother’s rejection of equal opportunities					
	*Equal opportunities*			ref.	
	*Better opportunities for Swiss*			0.320 **	0.116
Father’s rejection of equal opportunities					
	*Equal opportunities*			ref.	
	*Better opportunities for Swiss*			0.419 **	0.137
Composition household					
	*Adolescent living with two parents*	ref.		ref.	
	*Adolescent living with one parent*	-0.222	0.220	-0.203	0.227
	*Other household type*	-0.677	0.879	-0.395	0.258
	*Adolescent living alone*	0.359	1.079		
	*Adolescent living with partner and/or child*	-0.366	0.249		

Source: Swiss Household Panel (SHP), 1999–2017

Year-dummies included but not reported

N _model 1_ = 9,530 observations of 2,353 respondents; N _model 2_ = 8,948 observations of 2,125 respondents

*: p < 0.05,

**: p < 0.01,

***: p < 0.001 (tested two-tailed)

In the first model of “Table 3”, we also estimated the effect of the transition to vocational education on the likelihood to reject equal opportunities for foreigners. We found that those who made the transition to secondary vocational education did not significantly change in their rejection of equal opportunities. We did find a significant effect of the transition to tertiary vocational education, but this effect was opposed to what we expected. That is, we found that entering tertiary vocational education led to a decreased likelihood to reject equal opportunities instead of an increased likelihood. Additional analyses (see”S1 Text”) show that this negative significant effect is caused primarily by those who make the transition from secondary vocational training to tertiary vocational training, and not by those who make the transition from secondary education with Maturity. Considering that those who make the transition from secondary vocational training to tertiary vocational training already have had experience with vocational training and entering an apprenticeship, we could interpret this negative effect of tertiary vocational training on the change in rejection of equal opportunities as caused by entering a higher level of education and not per se as a result of entering an apprenticeship. All in all, we do not find support for our second hypothesis that states that entering an apprenticeship induces rejection of equal opportunities for foreigners.

Model 1 also includes the effect of adolescents’ financial dissatisfaction on negative attitudes towards equal opportunities for foreigners. Though we hypothesised that an increase in adolescent’s financial dissatisfaction would lead to an increased likelihood to support better opportunities for Swiss people, we found no significant effect. We performed an additional analysis to test whether results are different during times of economic recession, and found a significant effect of financial dissatisfaction on the likelihood to develop a negative attitude towards foreigners during the economic crisis (see”S2 Table”). Thus, those who became more dissatisfied with their economic situation during the recession, became more likely to reject equal opportunities for foreigners. Our third hypothesis is therefore not fully rejected.

### 4.3 Multivariate analyses on the impact of the household context

To test the impact of the household context, we focus only on adolescents who live with (one of) their parents. In the second model of “Table 3”, we removed those who do not live with their parents from our sample and included various household characteristics. A decrease in household income was expected to increase the likelihood to reject equal opportunities for foreigners. Mostly, we found effects that were statistically not significant. Compared to a change to the first income decile, we found that a change to the fourth and sixth income decile increased the likelihood to reject equal opportunities. Based on our hypothesis, we would, however, expect that the likelihood to reject equal opportunities would decrease as one’s household experiences a change towards a higher income decile compared to a change towards the lowest decile. Changing the reference category furthermore demonstrated that there was a positive effect of changing to the fourth category compared to changing to the second category, but no other significant effects of income were found when changing the reference categories. Taken together, these findings point at a lack of support for our fourth hypothesis.

Parental unemployment was also included in the second model to test whether a household’s confrontation with unemployment led to an increase in rejection of equal opportunities for foreigners among adolescents. The effect of parental unemployment was not significant. Again, the standard error was relatively high in comparison to all other effects. Moreover, our models rely here as well on a relatively low number of respondents that lived in a household that was confronted with a transition to unemployment (70 transitions to unemployment among (one of the) parents). This may indicate a lack of power, and therefore we cannot support or refute our fifth hypothesis. Moreover, our sixth hypothesis on the effect of changes in the household’s financial dissatisfaction is not supported. A change in a household’s financial dissatisfaction did not lead to an increased likelihood among adolescents to reject equal opportunities for foreigners.

To make sure that the absence of significant effects of the changes in economic household characteristics is not due to the inclusion of parents’ rejection of equal opportunities in this model, we also estimated a model in which we left out mothers’ and fathers’ attitudes towards equal opportunities for foreigners (not shown). This model does not show different outcomes as regards the effects of the economic household characteristics.

Additionally, we checked whether specific groups–adolescents who live in a household with a relatively lower income or whose parents have a relatively lower educational attainment–are to greater extent affected by a deterioration of the economic situation (see “S1 Appendix”). We expected that these specific groups were more likely to develop rejection of equal opportunities for foreigners as a result of a deteriorating income, a confrontation with unemployment, or an increase in financial dissatisfaction of the household. Also in these subgroups, changes in the household’s income, employment situation, or financial dissatisfaction did not evoke a significant change in adolescent’s likelihood to reject equal opportunities for foreigners. Robustness analyses showed that changing the cut-off points as to who is considered to live in household with a relatively lower income or with relatively lower educated parents did not yield different conclusions. Therefore, we reject hypothesis 7.

In the second model of “Table 3”, changes in mother’s and father’s rejection of equal opportunities are included as well. We found that a change in both mothers’ and fathers’ rejection of equal opportunities leads to a significant change in adolescents’ rejection of equal opportunities. As parents changed towards a negative stance towards equal opportunities, the likelihood for adolescents to reject equal opportunities increased concurrently.

Lastly, we performed a Bayesian analysis to reduce possible concerns regarding our rather small sample size for some categories [61]. Given the small number of adolescents and parents that made a transition to unemployment, we were particularly interested in the outcomes of this Bayesian analysis regarding our first and fifth hypothesis. The conclusions drawn from the Bayesian analysis (available upon request) are largely in line with the conclusions from our frequentist analysis as presented in “Table 3”. However, regarding adolescents’ transition to unemployment, we found a significant positive effect on the likelihood to reject equal opportunities for those living with their parents. Though we remain cautious considering our small N, this effect alludes to a change towards a greater likelihood to reject equal opportunities for foreigners due to unemployment, as was expected in Hypothesis 5. For parental unemployment, we found an effect in the opposite direction to what we expected. The results of this Bayesian analysis also point at support for our hypothesis regarding financial dissatisfaction within the household. Though we prefer to either support or refute hypotheses based on more conservative and stringent analyses, these Bayesian results provide us with additional insights from which we may derive that the null-hypothesis that there is no effect should not immediately accepted for the hypotheses that we found no support for in our initial analyses.

## 5. Conclusion and discussion

This study’s objective was to test the dynamic perspective of one of the key theoretical orientations in the literature on intergroup relations: realistic conflict theory. While it is argued that immigration rapidly changes the composition and face of European societies [7], only few studies have focused on how individuals’ intergroup attitudes change over this period. Considering that the few studies on this topic provide mixed evidence regarding the versatility of intergroup attitudes [see e.g. 8, 12, 15], we focused on changes in rejection of equal opportunities for foreigners among those who are, according to the ‘impressionable years’-hypothesis, presumed to be more likely to change in their attitudes: adolescents and young adults. By combining this notion from the psychological development literature with premises of realistic conflict theory, we aimed to contribute to the understanding of the dynamics of intergroup attitudes.

Firstly, we find, in contrast to some previous research [15, 17, 18], that intergroup attitudes are relatively stable among adolescents, just as they are among adults. The results of our study signify relatively high levels of stability in intergroup attitudes among citizens in general, adolescents included. Considering insights from Kustov, Laaker & Reller [15] who found that higher levels of stability of intergroup attitudes in panels with dichotomous variables as compared to ratio variables, our use of such a dichotomous dependent variable may have contributed to the high level of stability in rejection of equal opportunities for foreigners. Still, these findings could also imply that intergroup attitudes are already for a large part ‘crystallised’ before people reach late adolescence or young adulthood. Due to data limitations we were unable to examine changes in attitudes towards equal opportunities before the age of 14. Following the development of intergroup attitudes already from an even younger age onwards would be of help to find a more informative answer regarding the age of crystallisation of intergroup attitudes.

Nonetheless, still one in five adolescents changes their view on equal opportunities for foreigners substantially. We tried to explain such changes in rejection of equal opportunities among adolescents by formulating dynamic hypotheses derived from realistic conflict theory on the competitive situation of both the adolescent and the household that they lived in. Yet, hardly any support was found for our hypotheses on the impact of economic changes on the likelihood to reject equal opportunities.

In contrast to our expectations, the transition to employment did not increase but decrease rejection of equal opportunities. Because of power issues with few respondents making a transition to unemployment, we were not fully able to examine the role of the transition to unemployment on rejection of equal opportunities. However, additional Bayesian analyses point at an increased likelihood to reject equal opportunities because of a transition to unemployment among adolescents. Though we did not find evidence for our expectations regarding the presumed increase in rejection of equal opportunities as a result of the transition to the labour market, our results do not fully contradict presumptions of realistic conflict theory. Our result that points at a decreased likelihood of rejecting equal opportunities after finding a job might indicate that those who are able to find a more secure position on the labour market after leaving school are, because of finding a job, past the situation in which heightened levels of intergroup competition are perceived. Future research is, however, needed to substantiate this claim.

Moreover, we find that transitions to vocational education did not increase the likelihood to reject equal opportunities, which is in line with previous research [20]. It is therefore doubtful whether starting a vocational training evokes subjective perceptions of economic competition with foreigners among adolescents in Switzerland and other European countries. If this effect is already absent in Switzerland, a country with an educational system that is characterised by high numbers of students attending vocational training, it is questionable whether this effect is present at all in countries with educational systems that place less focus on vocational training.

What is furthermore striking is that there is no conclusive evidence for the influence of subjective economic changes on attitudes towards equal opportunities for foreigners. Though previous studies found subjective economic changes to be more influential than objective changes [11, 12], our study could not fully corroborate these findings. Especially in times of economic recession, when the economic situation is a more salient issue, we see that an increase in financial dissatisfaction among adolescents increases the likelihood to reject equal opportunities. Neither did we find unwavering support for the impact of changes on the household level in the subjective perception of the economic situation. We, thus, find that particularly in a context that is characterised by economic hardship, changes in subjective perceptions of financial dissatisfaction appeared to evoke a change in adolescents’ likelihood to reject equal opportunities.

Above all, the household context influenced changes in rejection of equal opportunities for foreigners among adolescents *directly* via changes in parents’ attitudes. Adolescents appeared to be affected less by changes in the household’s economic situations. Therewith, this study predominantly supports socialisation theories on parents’ direct influences rather than theories based on the ‘positional parental socialisation’-framework [e.g. 35, 36, 62]. Note that we did not aim to address the causality between the change in adolescents’ and their parents’ rejection of equal opportunities when testing our hypothesis on direct socialisation. Notwithstanding, it should be borne in mind that intergroup attitudes such as rejection of equal opportunities are often a result of mutual influences between adolescents and parents [23]. Caution regarding causality is also warranted for the various changes in economic situations that may be interrelated, but were beyond the scope of this study.

A limitation of this study is that we could not follow young adults in their transition from living in their parental home to living on their own and gaining more economic independence from their parents. For now, we acknowledged the economic dependence of young adults on their parents by including the (economic) household context, even though this context was only influential for rejection of equal opportunities via attitudinal socialisation.

All in all, our findings do not provide ubiquitous support for the dynamic perspective of realistic conflict theory. Despite of using a broad range of characteristics related to both adolescents’ and their household’s situation, changes in the economic situation hardly explain changes in rejection of equal opportunities for foreigners. Though this is not in line with some previous studies that contrarily demonstrate that changes in the economic situation do explain some changes in intergroup attitudes [e.g. 8, 20], it resonates and resembles findings of other studies that do not succeed in finding support for the dynamic effect of individuals’ economic situation [e.g. 9–11]. At the very least, our endeavour to contribute to the existing literature by shifting attention to adolescents and young adults corroborates the inconclusiveness of previous studies on the dynamic interpretation of realistic conflict theory. The impact of changes in one’s economic situation on within-person changes in intergroup attitudes is limited, both among the general population as well as among adolescents.

Since changing economic situations appear to be an ambiguous explanation of changing intergroup attitudes, more research is needed on why one in five adolescents still changes from offering foreigners equal opportunities to favouring Swiss citizens over foreigners or the other way around. Firstly, it is relevant to replicate this in other studies and in other countries, since Switzerland may be a special case with its non-membership of the EU and more restricted migration policies than its neighbouring countries. Secondly, employing a broader theoretical interpretation of experiencing threat might be useful as well. In this study, we mainly focused on changes in experienced or perceived economic competition that may deteriorate intergroup relations, while changes that may improve intergroup relations, for instance positive intergroup contact experiences [46], were not taken into account. But also within the domain of intergroup competition and threat, more insight can be gained on other factors that are not related to economic situations. Namely, if changes in the domain of the economy and perceptions about the financial situation cannot explain changes in rejection of equal opportunities for foreigners, we may need a shift of focus to non-economic conflict and (perceived) symbolic threats [63] that are to a lesser extent related to adolescents’ economic situation.

## Supporting information

S1 Table Different codings dependent variable. (DOCX)

S2 Table Logistic fixed effects analysis for economic recession (2007–2011). (DOCX)

S1 Text Transitions from different educational levels to employment. (DOCX)

S1 Appendix (DOCX)
