# Supplementary material for: Individual change in rejection of equal opportunities for foreigners among adolescents and young adults in Switzerland: Testing realistic conflict theory from a dynamic perspective
Source: PLoS One. 2024 Feb 7;19(2):e0296883. doi: 10.1371/journal.pone.0296883 (PMC10849248; doi:10.1371/journal.pone.0296883)
Supplement: S1 Table — (DOCX) [file pone.0296883.s001.docx]

**S1 Table. Different codings dependent variable**

| *Table S1.1: Logistic fixed effects analysis on the likelihood to reject equal opportunities for foreigners, category ‘neither’ is left out of the analyses* | | | | | | | |
| --- | --- | --- | --- | --- | --- | --- | --- |
|  |  | Model 1 | | | Model 2 | | |
|  |  | All adolescents | | | Adolescents who live with their parents | | |
|  |  | B | | S.E. | B | | S.E. |
|  |  |  | |  |  | |  |
| Labour market transitions | |  | |  |  | |  |
|  | Transition to employment | -0.509 * | | 0.164 | -0.390 * | | 0.170 |
|  | Transition to unemployment | 0.090 | | 0.471 | 0.129 | | 0.479 |
| Educational transitions | |  | |  |  | |  |
|  | Transition to secondary vocational | 0.158 | | 0.143 | 0.131 | | 0.146 |
|  | Transition to tertiary vocational | -0.664 ** | | 0.246 | -0.677 ** | | 0.252 |
| Financial dissatisfaction | | 0.007 | | 0.021 | 0.008 | | 0.022 |
|  |  |  | |  |  | |  |
| Household income | |  | |  |  | |  |
|  | *First decile* |  | |  | ref. | |  |
|  | *Second decile* |  | |  | 0.192 | | 0.221 |
|  | *Third decile* |  | |  | 0.446 | | 0.227 |
|  | *Fourth decile* |  | |  | 0.646 ** | | 0.231 |
|  | *Fifth decile* |  | |  | 0.503 * | | 0.235 |
|  | *Sixth decile* |  | |  | 0.619 ** | | 0.239 |
|  | *Seventh decile* |  | |  | 0.484 | | 0.248 |
|  | *Eighth decile* |  | |  | 0.495 | | 0.253 |
|  | *Ninth decile* |  | |  | 0.242 | | 0.266 |
|  | *Tenth decile* |  | |  | 0.615 * | | 0.286 |
| Unemployment parents | |  | |  | -0.399 | | 0.368 |
| Financial dissatisfaction household | |  | |  | 0.041 | | 0.030 |
| Mother's rejection of equal opportunities | |  | |  |  | |  |
|  | *Equal opportunities* |  | |  | ref. | |  |
|  | *Better opportunities for Swiss* |  | |  | 0.369 ** | | 0.124 |
| Father's rejection of equal opportunities | |  | |  |  | |  |
|  | *Equal opportunities* |  | |  | ref. | |  |
|  | *Better opportunities for Swiss* |  | |  | 0.437 ** | | 0.145 |
|  |  |  | |  |  | |  |
| Composition household | |  | |  |  | |  |
|  | *Adolescent living with two parents* | ref. | |  | ref. | |  |
|  | *Adolescent living with one parent* | -0.266 | | 0.220 | -0.273 | | 0.240 |
|  | *Other household type* | -0.380 | | 0.272 | -0.424 | | 0.283 |
|  | *Adolescent living alone* | -1.083 | | 0.969 |  | |  |
|  | *Adolescent living with partner and/or child* | 1.084 | | 1.294 |  | |  |
|  |  |  |  | |  |  | |
| *Source: Swiss Household Panel (SHP), 1999-2017*  *Year-dummies included but not reported*  *N _model 1_ = 8,955 observations of 2,345 respondents; N _model 2_ = 8,428 observations of 2,119 respondents*  **: p < 0.05, **: p < 0.01, ***: p < 0.001 (tested two-tailed)* | | | | | | | |

| *Table S1.2: Logistic fixed effects analysis on the likelihood to reject equal opportunities for foreigners, category ‘neither’ is ascribed a score of 1 instead of 0* | | | | | | | |
| --- | --- | --- | --- | --- | --- | --- | --- |
|  |  | Model 1 | | | Model 2 | | |
|  |  | All adolescents | | | Adolescents who live with their parents | | |
|  |  | B | | S.E. | B | | S.E. |
|  |  |  | |  |  | |  |
| Labour market transitions | |  | |  |  | |  |
|  | Transition to employment | -0.283 * | | 0.144 | -0.270 | | 0.150 |
|  | Transition to unemployment | 0.086 | | 0.416 | 0.167 | | 0.421 |
| Educational transitions | |  | |  |  | |  |
|  | Transition to secondary vocational | 0.137 | | 0.128 | 0.136 | | 0.131 |
|  | Transition to tertiary vocational | -0.394 | | 0.209 | -0.412 | | 0.212 |
| Financial dissatisfaction | | 0.011 | | 0.019 | 0.014 | | 0.020 |
|  |  |  | |  |  | |  |
| Household income | |  | |  |  | |  |
|  | *First decile* |  | |  | ref. | |  |
|  | *Second decile* |  | |  | 0.119 | | 0.191 |
|  | *Third decile* |  | |  | 0.303 | | 0.196 |
|  | *Fourth decile* |  | |  | 0.418 * | | 0.199 |
|  | *Fifth decile* |  | |  | 0.244 | | 0.202 |
|  | *Sixth decile* |  | |  | 0.335 | | 0.206 |
|  | *Seventh decile* |  | |  | 0.259 | | 0.214 |
|  | *Eighth decile* |  | |  | 0.212 | | 0.217 |
|  | *Ninth decile* |  | |  | 0.073 | | 0.228 |
|  | *Tenth decile* |  | |  | 0.304 | | 0.246 |
| Unemployment parents | |  | |  | -0.242 | | 0.311 |
| Financial dissatisfaction household | |  | |  | 0.016 | | 0.026 |
| Mother's rejection of equal opportunities | |  | |  |  | |  |
|  | *Equal opportunities* |  | |  | ref. | |  |
|  | *Better opportunities for Swiss* |  | |  | 0.333 ** | | 0.108 |
| Father's rejection of equal opportunities | |  | |  |  | |  |
|  | *Equal opportunities* |  | |  | ref. | |  |
|  | *Better opportunities for Swiss* |  | |  | 0.264 * | | 0.128 |
|  |  |  | |  |  | |  |
| Composition household | |  | |  |  | |  |
|  | *Adolescent living with two parents* | ref. | |  | ref. | |  |
|  | *Adolescent living with one parent* | -0.142 | | 0.205 | -0.144 | | 0.212 |
|  | *Other household type* | -0.102 | | 0.214 | -0.090 | | 0.223 |
|  | *Adolescent living alone* | -0.606 | | 0.691 |  | |  |
|  | *Adolescent living with partner and/or child* | -0.032 | | 0.753 |  | |  |
|  |  |  |  | |  |  | |
| *Source: Swiss Household Panel (SHP), 1999-2017*  *Year-dummies included but not reported*  *N _model 1_* *= 9,530 observations of 2,353 respondents; N _model 2_ = 8,948 observations of 2,125 respondents*  **: p < 0.05, **: p < 0.01, ***: p < 0.001 (tested two-tailed)* | | | | | | | |
