# Supplementary material for: Individual change in rejection of equal opportunities for foreigners among adolescents and young adults in Switzerland: Testing realistic conflict theory from a dynamic perspective
Source: PLoS One. 2024 Feb 7;19(2):e0296883. doi: 10.1371/journal.pone.0296883 (PMC10849248; doi:10.1371/journal.pone.0296883)
Supplement: S2 Table — (DOCX) [file pone.0296883.s002.docx]

**S2 Table. Logistic fixed effects analysis for economic recession (2007-2011)**

| *Table S2: Logistic fixed effects analysis on the likelihood to reject equal opportunities for foreigners during the economic recession (2007-2011)* | | | | | |
| --- | --- | --- | --- | --- | --- |
|  |  | Model 1 | | Model 2 | |
|  |  | All adolescents | | Adolescents who live with their parents | |
|  |  | B | S.E. | B | S.E. |
|  |  |  |  |  |  |
| Labour market transitions | |  |  |  |  |
|  | Transition to employment | -0.895 * | 0.418 | -1.103 * | 0.445 |
|  | Transition to unemployment | 14.525 | 1218.574 | 13.523 | 574.347 |
| Educational transitions | |  |  |  |  |
|  | Transition to secondary vocational | 0.295 | 0.305 | 0.292 | 0.315 |
|  | Transition to tertiary vocational | 0.927 | 0.601 | 0.794 | 0.642 |
| Financial dissatisfaction | | 0.122 ** | 0.046 | 0.118 * | 0.048 |
|  |  |  |  |  |  |
| Household income | |  |  |  |  |
|  | *First decile* |  |  | ref. |  |
|  | *Second decile* |  |  | 0.655 | 0.567 |
|  | *Third decile* |  |  | 0.008 | 0.586 |
|  | *Fourth decile* |  |  | 0.678 | 0.572 |
|  | *Fifth decile* |  |  | 0.526 | 0.566 |
|  | *Sixth decile* |  |  | 0.684 | 0.580 |
|  | *Seventh decile* |  |  | 0.409 | 0.599 |
|  | *Eighth decile* |  |  | 0.674 | 0.590 |
|  | *Ninth decile* |  |  | 0.352 | 0.613 |
|  | *Tenth decile* |  |  | 0.981 | 0.660 |
| Unemployment parents | |  |  | 1.281 | 0.739 |
| Financial dissatisfaction household | |  |  | 0.024 | 0.060 |
| Mother's rejection of equal opportunities | |  |  |  |  |
|  | *Equal opportunities* |  |  | ref. |  |
|  | *Better opportunities for Swiss* |  |  | 0.274 | 0.277 |
| Father's rejection of equal opportunities | |  |  |  |  |
|  | *Equal opportunities* |  |  | ref. |  |
|  | *Better opportunities for Swiss* |  |  | 0.512 | 0.324 |
|  |  |  |  |  |  |
| Composition household | |  |  |  |  |
|  | *Adolescent living with two parents* |  |  |  |  |
|  | *Adolescent living with one parent* | 0.276 | 0.580 | 0.025 | 0.612 |
|  | *Other household type* | 0.616 | 1.724 | -0.069 | 0.791 |
|  | *Adolescent living alone* | 14.347 | 1145.448 |  |  |
|  | *Adolescent living with partner and/or child* | -0.028 | 0.746 |  |  |
|  |  |  |  |  |  |
| *Source: Swiss Household Panel (SHP), 2007-2011*  *Year-dummies included but not reported*  *N _model 1_ = 2,992 observations of 1,195 respondents; N _model 2_ = 2,877 observations of 1,137 respondents*  **: p < 0.05, **: p < 0.01, ***: p < 0.001 (tested two-tailed)* | | | | | |
