# Supplementary material for: Individual change in rejection of equal opportunities for foreigners among adolescents and young adults in Switzerland: Testing realistic conflict theory from a dynamic perspective
Source: PLoS One. 2024 Feb 7;19(2):e0296883. doi: 10.1371/journal.pone.0296883 (PMC10849248; doi:10.1371/journal.pone.0296883)
Supplement: S1 Text — (DOCX) [file pone.0296883.s003.docx]

**S1 Text. Transitions from different educational levels to employment**

In this appendix, we elaborate on the way in which various labour market and educational transitions affect changes in attitudes towards equal opportunities for foreigners. To get an insight in the effects of these transitions, we estimated a model in which we included the transition to employment and unemployment for each educational level (see Table 1). We also estimated the effect of the most usual transitions in education on rejection of equal opportunities. We do not report the effects of the transition from primary education to (un)employment, due to irregular patterns in the panel data. Also, note that in some cases only very few respondents were classified as having experienced such a transition. For instance, only seven respondents experienced the transition from tertiary vocational training to unemployment. Due to these low number of respondents experiencing several transitions, some of the effects could not be estimated or led to unreliable B-coefficients with very high standard errors. Therefore, these results should be interpreted with caution.

In the results section we found that the transition from school to employment decreased the likelihood to take a negative attitude towards equal opportunities when not controlling for the impact of the household context. When breaking down the transitions to employment and unemployment for different educational levels, we do, however, not find a significant of one of the transitions to employment. It is therefore likely that the negative effect of entering employment on the change in adolescents’ attitude towards equal opportunities is driven by transitions from one or more particular educational levels to employment.

Though we did not formulate specific hypotheses on the educational transitions, we also estimated a model in which we test which transitions in education led to a significant change in the likelihood to reject equal opportunities. We find that the negative effect of the transition to tertiary vocational education on rejection of equal opportunities is mainly driven by those who enter tertiary vocational education after being in secondary vocational education. Entering tertiary vocational education after being in secondary education with Maturity did not lead to a significant change in the likelihood to reject equal opportunities. Furthermore, we found that the transition from secondary education with Maturity to university led to a significant increase in the likelihood to reject equal opportunities. All other educational transitions did not have a significant impact on the likelihood to reject equal opportunities for foreigners.

We also estimated a model in which we also included the household characteristics (results available upon request). The transitions that had a significant effect on rejection of equal opportunities in Model 1 did not change when controlling for these household characteristics.

| *Table S1: Logistic fixed effects analysis on the likelihood to reject equal opportunities for foreigners* | | | |
| --- | --- | --- | --- |
|  |  | Model 1 | |
|  |  | B | S.E. |
|  |  |  |  |
| Labour market transitions | |  |  |
|  | Secondary, no Maturity > Employment | -0.876 | 1.445 |
|  | Secondary, no Maturity > Unemployment |  |  |
|  | Secondary, with Maturity > Employment | 0.572 | 0.517 |
|  | Secondary, with Maturity > Unemployment | -0.134 | 0.939 |
|  | Secondary vocational > Employment | -0.261 | 0.173 |
|  | Secondary vocational > Unemployment | 0.173 | 0.541 |
|  | Tertiary vocational > Employment | -0.945 | 0.539 |
|  | Tertiary vocational > Unemployment | 14.363 | 871.854 |
|  | University > Employment | -0.001 | 0.677 |
|  | University > Unemployment |  |  |
| Educational transitions | |  |  |
|  | Primary > Secondary, no Maturity | 0.572 | 0.650 |
|  | Primary > Secondary, with Maturity | 0.006 | 0.238 |
|  | Primary > Secondary vocational | 0.229 | 0.150 |
|  | Secondary, with Maturity > Tertiary vocational | -0.449 | 0.365 |
|  | Secondary, with Maturity > University | 0.509 * | 0.240 |
|  | Secondary vocational > Tertiary vocational | -0.878 | 0.326 |
| Financial dissatisfaction | | 0.020 | 0.020 |
|  |  |  |  |
| Composition household | |  |  |
|  | *Adolescent living with two parents* | ref. |  |
|  | *Adolescent living with one parent* | -0.287 | 0.223 |
|  | *Other household type* | -0.356 | 0.251 |
|  | *Adolescent living alone* | -0.506 | 0.901 |
|  | *Adolescent living with partner and/or child* | 0.514 | 1.104 |
|  |  |  |  |
| *Source: Swiss Household Panel (SHP), 1999-2017*  *Year-dummies included but not reported*  *N = 9,530 observations of 2,353 respondents*  **: p < 0.05, **: p < 0.01, ***: p < 0.001 (tested two-tailed)* | | | |
