# Supplementary material for: Individual change in rejection of equal opportunities for foreigners among adolescents and young adults in Switzerland: Testing realistic conflict theory from a dynamic perspective
Source: PLoS One. 2024 Feb 7;19(2):e0296883. doi: 10.1371/journal.pone.0296883 (PMC10849248; doi:10.1371/journal.pone.0296883)
Supplement: S1 Appendix — (DOCX) [file pone.0296883.s004.docx]

**Appendix**

| *Table A.1: Logistic fixed effects analysis on the likelihood to reject equal opportunities for foreigners among adolescents living in a household with a relatively low income* | | | | | |
| --- | --- | --- | --- | --- | --- |
|  |  | Model 1 | | Model 2 | |
|  |  | B | S.E. | B | S.E. |
|  |  |  |  |  |  |
| Labour market transitions | |  |  |  |  |
|  | Transition to employment | -0.037 | -0.140 | -0.024 | 0.270 |
|  | Transition to unemployment | -0.063 | -0.090 | -0.147 | 0.749 |
| Educational transitions | |  |  |  |  |
|  | Transition to secondary vocational | 0.071 | 0.340 | 0.042 | 0.208 |
|  | Transition to tertiary vocational | -0.712 | -1.750 | -0.768 | 0.416 |
| Financial dissatisfaction | | -0.005 | -0.150 | -0.007 | 0.033 |
|  |  |  |  |  |  |
| Household income | |  |  |  |  |
|  | *First decile* |  |  | ref. |  |
|  | *Second decile* |  |  | 0.104 | 0.217 |
|  | *Third decile* |  |  | 0.258 | 0.230 |
|  | *Fourth decile* |  |  | 0.264 | 0.247 |
|  | *Fifth decile* |  |  | 0.213 | 0.278 |
|  | *Sixth decile* |  |  | 0.389 | 0.309 |
|  | *Seventh decile* |  |  | 0.539 | 0.439 |
|  | *Eighth decile* |  |  | 0.073 | 0.469 |
|  | *Ninth decile* |  |  | -0.540 | 0.856 |
|  | *Tenth decile* |  |  | 0.484 | 1.475 |
| Unemployment parents | |  |  | -0.508 | 0.453 |
| Financial dissatisfaction household | |  |  | 0.063 | 0.040 |
| Mother's rejection of equal opportunities | |  |  |  |  |
|  | *Equal opportunities* |  |  | ref. |  |
|  | *Better opportunities for Swiss* |  |  | 0.250 | 0.185 |
| Father's rejection of equal opportunities | |  |  |  |  |
|  | *Equal opportunities* |  |  | ref. |  |
|  | *Better opportunities for Swiss* |  |  | 0.193 | 0.211 |
|  |  |  |  |  |  |
| Composition household | |  |  |  |  |
|  | *Adolescent living with two parents* | ref*.* |  | ref. |  |
|  | *Adolescent living with one parent* | -0.091 | -0.260 | -0.145 | 0.365 |
|  | *Other household type* | -0.435 | -1.130 | -0.389 | 0.392 |
|  |  |  |  |  |  |
| *Source: Swiss Household Panel (SHP), 1999-2017*  *Year-dummies included but not reported*  *N = 3,402 observations of 822 respondents*  **: p < 0.05, **: p < 0.01, ***: p < 0.001 (tested two-tailed)* | | | | | |

| *Table A.2: Logistic fixed effects analysis on the likelihood to reject equal opportunities for foreigners among adolescents whose parents have a relatively low educational attainment* | | | | | |
| --- | --- | --- | --- | --- | --- |
|  |  | Model 1 | | Model 2 | |
|  |  | B | S.E. | B | S.E. |
|  |  |  |  |  |  |
| Labour market transitions | |  |  |  |  |
|  | Transition to employment | -0.456 * | 0.217 | -0.489 * | 0.226 |
|  | Transition to unemployment | 0.177 | 0.636 | 0.267 | 0.661 |
| Educational transitions | |  |  |  |  |
|  | Transition to secondary vocational | -0.020 | 0.197 | -0.039 | 0.201 |
|  | Transition to tertiary vocational | -0.696 | 0.391 | -0.680 | 0.397 |
| Financial dissatisfaction | | 0.015 | 0.029 | 0.016 | 0.030 |
|  |  |  |  |  |  |
| Household income | |  |  |  |  |
|  | *First decile* |  |  | ref. |  |
|  | *Second decile* |  |  | 0.121 | 0.249 |
|  | Third decile |  |  | -0.005 | 0.270 |
|  | *Fourth decile* |  |  | 0.457 | 0.279 |
|  | *Fifth decile* |  |  | 0.107 | 0.287 |
|  | *Sixth decile* |  |  | 0.539 | 0.294 |
|  | *Seventh decile* |  |  | 0.197 | 0.327 |
|  | *Eighth decile* |  |  | 0.085 | 0.328 |
|  | *Ninth decile* |  |  | -0.169 | 0.375 |
|  | *Tenth decile* |  |  |  |  |
| Unemployment parents | |  |  | -0.322 | 0.470 |
| Financial dissatisfaction household | |  |  | 0.050 | 0.039 |
| Mother's rejection of equal opportunities | |  |  |  |  |
|  | *Equal opportunities* |  |  | ref. |  |
|  | *Better opportunities for Swiss* |  |  | 0.441 ** | 0.171 |
| Father's rejection of equal opportunities | |  |  |  |  |
|  | *Equal opportunities* |  |  | ref. |  |
|  | *Better opportunities for Swiss* |  |  | 0.133 | 0.201 |
|  |  |  |  |  |  |
| Composition household | |  |  |  |  |
|  | *Adolescent living with two parents* | ref. |  | ref. |  |
|  | *Adolescent living with one parent* | -0.223 | 0.334 | -0.142 | 0.340 |
|  | *Other household type* | -0.600 | 0.392 | -0.529 | 0.410 |
|  |  |  |  |  |  |
| *Source: Swiss Household Panel (SHP), 1999-2017*  *Year-dummies included but not reported*  *N = 3,606 observations of 934 respondents*  **: p < 0.05, **: p < 0.01, ***: p < 0.001 (tested two-tailed)* | | | | | |
